# RNA-binding proteins in ovarian cancer: a novel avenue of their roles in diagnosis and treatment

**DOI:** 10.1186/s12967-022-03245-6

**Published:** 2022-01-21

**Authors:** Jiangchun Wu, Yong Wu, Qinhao Guo, Simin Wang, Xiaohua Wu

**Affiliations:** 1 Department of Oncology, Shanghai Medical College, Fudan University, Shanghai, 200032 China; 2 Department of Gynecologic Oncology, Fudan University Shanghai Cancer Center, Fudan University, Shanghai, 200032 People’s Republic of China

**Keywords:** Ovarian cancer, RNA-binding proteins, Therapeutic targets

## Abstract

Ovarian cancer (OC), an important cause of cancer-related death in women worldwide, is one of the most malignant cancers and is characterized by a poor prognosis. RNA-binding proteins (RBPs), a class of endogenous proteins that can bind to mRNAs and modify (or even determine) the amount of protein they can generate, have attracted great attention in the context of various diseases, especially cancers. Compelling studies have suggested that RBPs are aberrantly expressed in different cancer tissues and cell types, including OC tissues and cells. More specifically, RBPs can regulate proliferation, apoptosis, invasion, metastasis, tumorigenesis and chemosensitivity and serve as potential therapeutic targets in OC. Herein, we summarize what is currently known about the biogenesis, molecular functions and potential roles of human RBPs in OC and their prospects for application in the clinical treatment of OC.

## Introduction

Ovarian cancer (OC) has been reported to be the fifth leading cause of death among females, and an estimated 21,410 new cancer cases and 13,770 cancer-related deaths occurred in the United States in 2020 [1]. Most patients are diagnosed at an advanced stage of disease due to the deep anatomical position in the pelvic cavity and the lack of specific diagnostic symptoms or biomarkers [2]. Standard treatment, including surgery and chemotherapy, is usually effective at inducing remission, but in 70–80% of patients, the cancer recurs within 2 years [3]. OC is characterized by advanced stage diagnosis, rapid progression, high metastasis and recurrence rates, and rapid drug resistance development [4]. Despite numerous efforts to improve the efficacy of surgery, chemoradiotherapy, and targeted treatments, such as antiangiogenic drugs and poly (ADP-ribose) polymerase inhibitors, few reliable biomarkers or notably better therapeutic strategies for treating OC in daily clinical practice have been discovered in recent years [5].

RNA-binding proteins (RBPs) are proteins that play critical roles in the regulation of many RNA transcripts at multiple posttranscriptional levels [6]. Several studies have demonstrated that RBPs are abnormally expressed in cancer tissues relative to adjacent normal tissues, and this expression is related to patient prognosis [7–9]. Studies by our research group revealed a significant role of CUGBP- and ETR-3-like family 2 (CELF2) and Lin28 homologue B (LIN28B) in OC progression, and our interest in RBPs has grown [10]. Therefore, summarizing the functions and mechanisms of RBPs may broaden the field of OC research.

## Biogenesis and domain features of RBPs

### Biogenesis

**Fig. 1:**
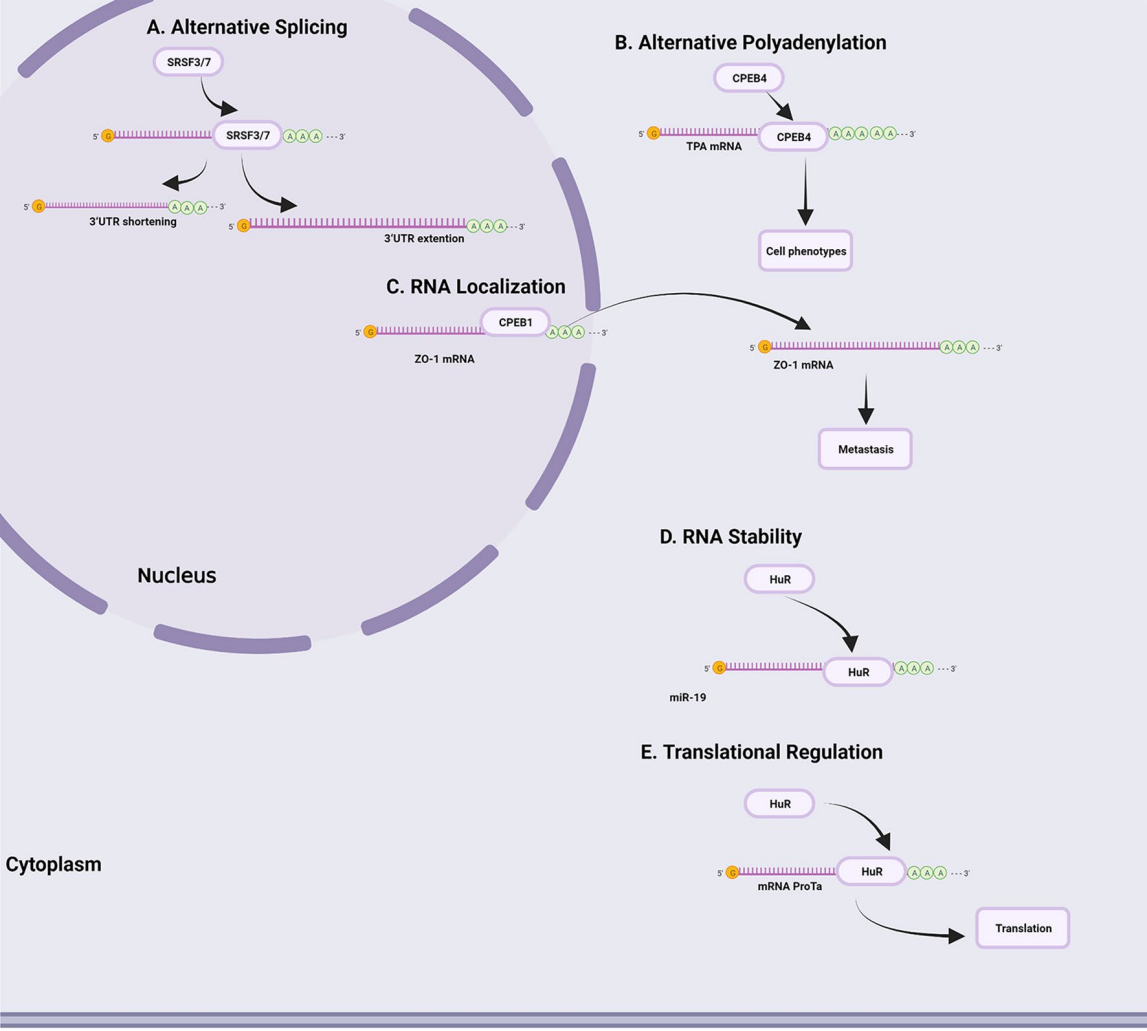
Various mechanisms regulate changes in RBP expression in cancer cells. **A** Genomic control. **B** Transcriptional regulation. **C** Posttranscriptional regulation. **D** Posttranslational modifications

RBPs are proteins that bind at specific target sites and impact the expression of coordinated sets of mRNAs [11]. The abnormal expression of RBPs has been widely reported in multiple types of cancer cells and is regulated by numerous mechanisms, including genomic control, transcriptional regulation, posttranscriptional modification (PTM), and posttranslational modification [12] (Fig. 1).

Genomic mutations, a prevalent feature of cancer cells, appear to have a minor contribution to abnormal RBP expression. Mutations in genes encoding RBPs are particularly rare compared to those in other genes [13, 14]. Somatic mutations, especially in spliceosome genes, are associated with haematopoietic disorders that result in acute myeloid leukaemia [15, 16]. Germline mutations in the DICER1 gene, which encodes a cytoplasmic endoribonuclease that processes precursor messenger RNAs (pre-mRNAs) into mature mRNAs, are reported for less than 1% of RBPs by the Online Mendelian Inheritance in Man database [16–18]. In addition, chromosomal translocations involving RBPs are relatively rare in cancer. In contrast, copy number variations are more common and are related to the deregulation of RBP expression [19].

RBP expression deregulation is also driven by transcriptional alterations [20, 21]. For example, RBP38 expression can be induced by the tumour suppressor P53, which in turn mediates the stabilization of P53 downstream target mRNAs that promote cell cycle arrest in the G1 phase [22, 23]. Posttranscriptional alteration is another mechanism involved in RBP expression deregulation [24]. The expression of the RBP Musashi 1 (MSI1) can be repressed by a class of tumour suppressor miRNAs, including miR-34a, miR-138, and miR-137, inhibiting the proliferation of glioblastoma and medulloblastoma cells [25, 26]. Recent studies have emphasized the role of translational modification in RBP expression deregulation. RBPs are hotspots for posttranscriptional modifications (PTMs), including acetylation, phosphorylation, methylation, and ubiquitination [27, 28]. For example, the acetylation of the RBP SAM68 can enhance its binding to mRNA and play a critical role in cell cycle control [29].

Overall, these studies provide an overview of the changes in RBPs involved in various mechanisms that may be therapeutic targets in the future.

### Domain features of RBPs

Through next‐generation sequencing (NGS) of diverse tumour cell types, a catalogue of 1542 experimentally validated human RBPs representing approximately 7.5% of all protein-coding genes in the genome was generated [30, 31]. Since RBPs play multiple biological roles, their structures comprise multiple small domains, including RNA recognition domains and RNA-binding domains interspersed between catalytic domains that enable them to recognize a wide range of downstream targets and regulate their catalytic activities [32]. These catalytic domains include three parts: deaminases, RNAse III domains and helicases. Multiple RNA-binding domains (RBDs) can specifically recognize and bind to RNA sequences [33]. Among the RBPs represented, a quarter contain conventional RBDs, and the rest contain nonconventional RBDs. Conventional RBDs may comprise RNA recognition motifs (RRMs), zinc fingers, S1 domains, PIWI domains, double-stranded RNA-binding domains (dsRBDs), K-homology (KH) domains, PIWI, AGO, and Zwill (PAZ) domains, while nonconventional RBPs have internally disordered regions and adjust their spatial structure to bind to RNAs and subsequently mediate cell regulation, signal transduction and metabolism [34].

## Functions of RBPs in cancers

**Fig. 2:**
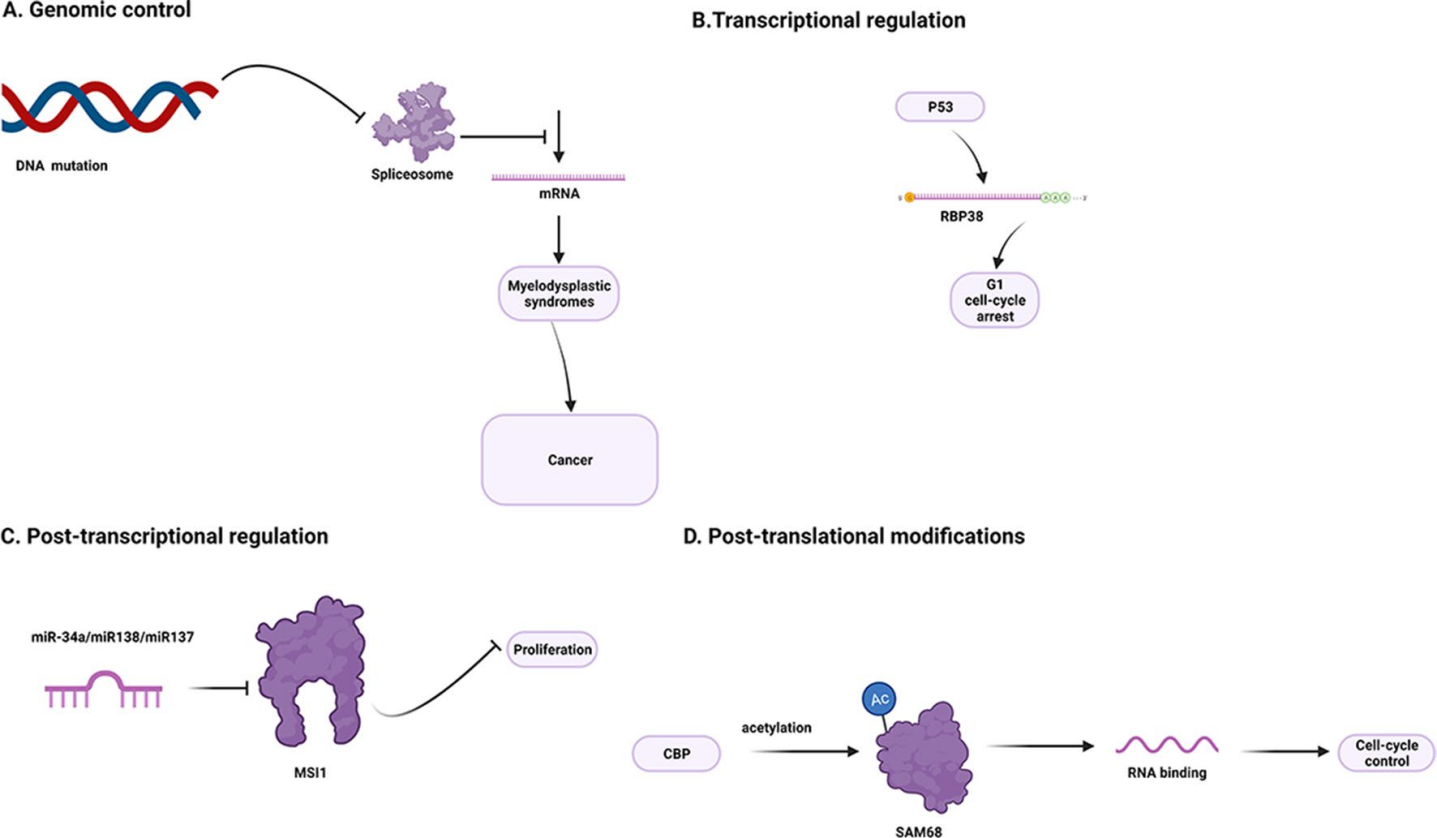
RBPs regulate the functions of transcripts at multiple post-transcriptional levels. **A** Alternative Splicing. **B** Alternative Polyadenylation. **C** RNA Localization. **D** RNA Stability. **E** Alternative Splicing

Because RBPs regulate various downstream targets in omnidirectional and multifunctional manners, even small alterations in their expression or activity can cause significant changes in regulatory networks. RBPs can interact with proteins or multiple RNAs, including mRNAs and ncRNAs, to form RNP complexes [35] and subsequently regulate the functions of RNA transcripts via multiple posttranscriptional mechanisms, including RNA splicing, polyadenylation, effects on localization and stability, and translational modification [35, 36] (Fig. 2).

Alternative splicing, a fundamental posttranscriptional regulation mechanism, contributes to the complexity and diversity of the proteome, resulting in changes in cancer cell phenotype [37, 38]. The regulation of alterative splicing relies on RBPs, which act as splicing factors that precisely bind to RNA motifs located in exons or their adjacent introns [38, 39]. The serine/arginine-rich (SR) protein and hnRNP families are essential RBPs that play evolutionarily conserved roles as regulators of alterative pre-mRNA splicing [40]. SRSF3 and SRSF7 have been shown to bind different sites in terminal exons and recruit NXF1 to regulate the length of the 3’ untranslated region (UTR) [41].

Alterative polyadenylation is another process that is essential for generating mature RNA transcripts. It occurs in 3’ UTRs, resulting in different lengths by cleavage and polyadenylation (CPA). The function of the 3’-UTR is essential for mRNA maturation, stability, localization, and translation. RBPs can either recruit or compete with polyadenylation machinery proteins, which regulate the cleavage and polyadenylation (CPA) of target mRNAs. In pancreatic cancer, CPEB4 regulates tail elongation with TPA mRNA, resulting in alteration of cancer cell proliferation, migration, invasion and angiogenesis [42].

RBPs also play a key role in subcellular localization by binding to the 3’ UTR sequence of mRNA or other ncRNAs, nucleating the assembly of multisubunit complexes that link transcripts to cytoskeletal motors that send ribonucleoproteins (RNPs) to specific subcellular locations [43]. CPEB1 encodes an essential tight junction component that regulates the localization of ZO-1 mRNA. Its depletion may impair cell polarity, resulting in alteration of metastatic potential and EMT-related genes in breast cancer [44, 45].

RBPs also play a key role in the stabilization or destabilization of specific target mRNAs or ncRNAs. The stability of RNAs is determined by their 3’ poly(A) tail and 5’-terminal 7-methylguanosine (m7G). HuR (a member of the Hu family of RBPs)-mediated mRNA stabilization relies on the subcellular localization of HuR, which is translocated into the cytoplasm and is associated with cell cycle regulators and inflammation. For example, HuR can stabilize mRNAs that encode cyclins (A, B1, D1 and E), HIF-1a, and VEGF, thus increasing the expression of these proteins. In contrast, the mRNA levels of c-Myc and WNT-5A can be destabilized by HuR in cancer cells [35, 46–48].

RBPs are involved in multiple steps of translation, such as initiation, elongation, and termination, forming RNP complexes. A large number of related RBPs bind to the 5’ or 3’ UTR, resulting in different translation efficiencies [49]. HuR increases the abundance and translation of ProTa mRNA by targeting its 3’ UTR, which has been correlated with cancer progression [50].

## Profiles of RBP expression in OC

**Fig. 3:**
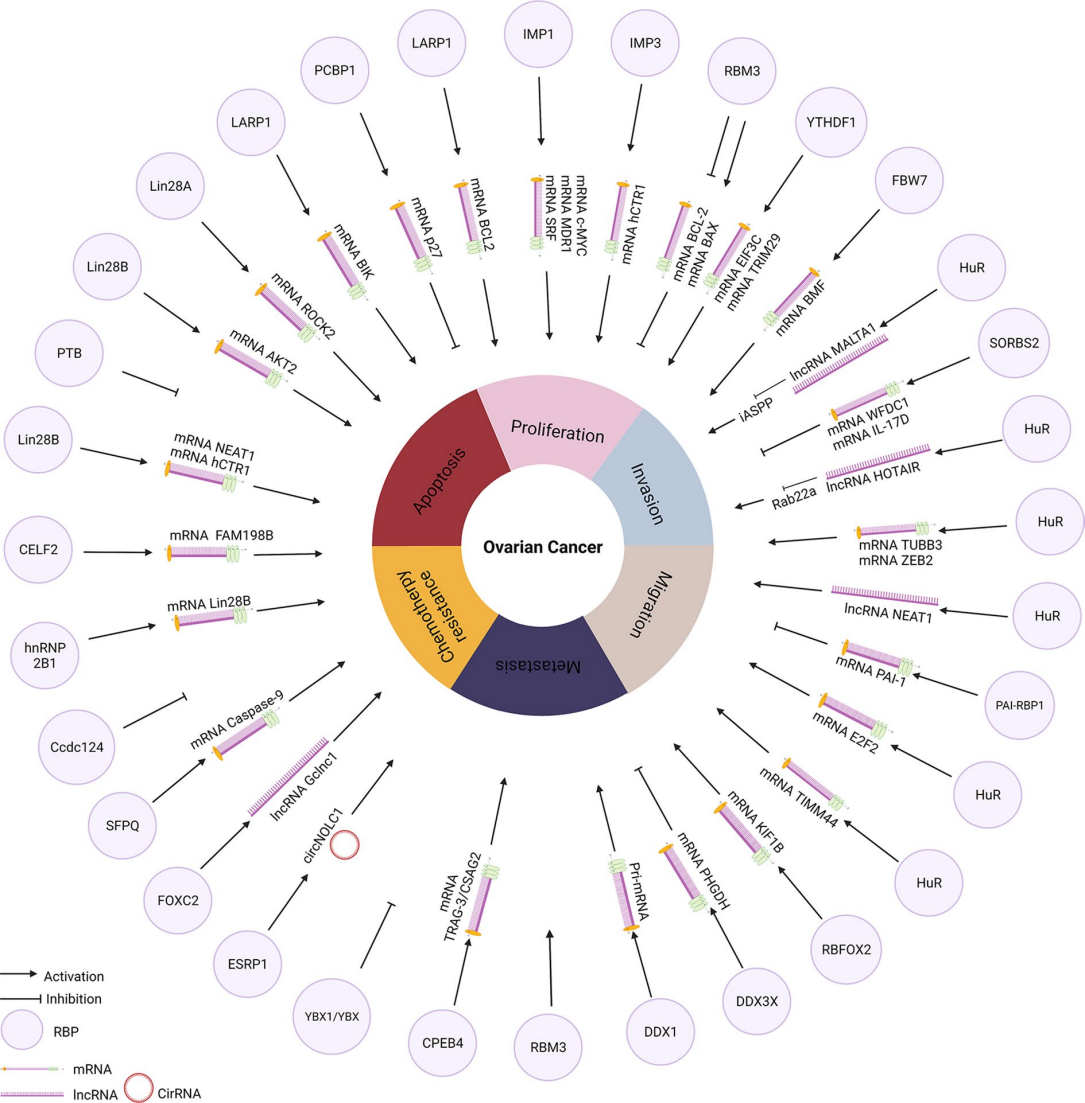
RBPs act as oncogenes or tumour suppressors in OC

Abnormal RBP expression can lead to genome-wide changes in the transcriptome and proteome levels and subsequently affect cell proliferation, apoptosis, angiogenesis, senescence, epithelial-mesenchymal transition (EMT), invasion, and metastasis [6]. Therefore, it is not surprising that altered expression of RBPs is common during the development and progression of cancers. Next, the alteration of RBP expression in OC is summarized (Table 1), and related phenotype changes are also presented (Fig. 3).

**Table 1:** Experimental evidence of altered RBPs in OC

Oncogenes					
RBP		Mechanism	Putative targets	Cellular phenotype	References
Insulin-like growth factor 2 mRNA-Binding Protein	IGF2BP1/IMP1	mRNA stability, Inhibiting decay	c-MYC, MDR1, SRF	Platinum, Chemoresistance, Growth, invasion	[101]
	IGF2BP2/IMP2			Proliferation	[90, 125]
	IGF2BP3/IMP3	mRNA stability, mRNA translation	HCTR1	Platinum, Chemoresistance, Proliferation, Migration, Invasion	[70]
La-Related Protein 1	LARP1	mRNA stability, mRNA de-stability	BIK, BCL2	Proliferation	[67]
Lin28	Lin28A	Alternative splicing, mRNA translation	ROCK2	Invasion, Metastasis, Proliferation, Migration, Invasion	[84]
	LIN28B	mRNA translation	AKT2, NEAT1	Platinum Chemoresistance, Proliferation, Migration, Invasion	[70]
Heterogeneous nuclear ribonucleoproteins (hnRNPs)	hnRNPA2B1	mRNA stability	Lin28B	Proliferation, Invasion, Migration, Apoptosis	[69]
Hu family of RBPs (ELAV-like protein 1)	HuR	mRNA stability, mRNA translation	TUBB3, lncRNA NEAT1, lncRNA MALAT1, lncRNA HOTAIR, ZEB2, TP53, E2F2	Proliferation, Invasion, Migration, Paclitaxel resistance	[60, 64, 91–93]
Y-box binding protein 1	YB1	mRNA stability, Translation modification		Chemotherapy resistance	[106]
YT521-B homology (YTH) domain-containing proteins	YTHDF1	mRNA translation	EIF3C, TRIM29	Chemotherapy resistance	[56, 126]
	YTHDF2	mRNA translation	BMF	Proliferation, Migration, Invasion, Apoptosis	[58]
Polypyrimidine-tract binding protein-associated splicing factor	SFPQ	Alternative splicing	Caspase-9	Apoptosis	[70]
Forkhead box protein C2	FOXC2		GClnc1	EMT	[95]
epithelial cell RBP	ESRP1	Alternative splicing	Circ-NOLC1	Prognosis	[72]
*Tumour suppressors*					
RNA-binding motif protein 3	RBM3	Translation	BCL-2, BAX	Platinum sensitivity	[3, 77]
CUGBP and ETR-3-like family 2	CELF2	mRNA stability	FAM198B	Proliferation, Migration, Invasion	[10]
Poly C Binding Protein 1	PCBP1	translational	p27	Inhibiting OC progress	[78]
heterogeneous nuclear family (hnRNPI)	PTB	Alternative splicing			[88]
Coiled-coil domain containing protein-124	Ccdc124			Prognosis	[79]
Y-box binding protein 1	YBX1/YB1			Prognosis, resistance to cisplatin	[106]
DEAD-Box Helicase 3X-Linked	DDX3X	Translation	PHGDH		[80]
PAI-1 mRNA Binding Protein 1	PAI-RBP1		PAI-1	Progression	[83]
Sorbin and SH3 domain-containing 2	SORBS2		WFDC1, IL-17D	Cell migration, Metastatic	[99]
RNA-binding motif protein 3	RBM3	DNA damage, Repair adverse cytotoxic effects after chemotherapy	BCL-2, BAX, DNA integrity	Prognosis, Platinum sensitivity	[3, 77]
Quaking isoforms 5	QKI5	stability	TAZ	inhibit metastasis	[100]

## RBPs act as oncogenes or tumour suppressors in modulating OC phenotypes

### Proliferation

Insulin-like growth factor 2 mRNA-binding protein 1 (IGF2BP1/IMP1), a member of the IMP family, binds to the 5’ UTR of IGF2 mRNA [51]. IGF2BP1 has the most conserved “oncogenic” role in the IGF2BP family and is associated with poor prognosis [52]. Serum response factor (SRF)-encoding mRNA acts as a downstream target of IGF2BP1, which destabilizes cell identity [53]. IMP1 is overexpressed in high serous ovarian carcinoma (HGSOC), acts as a novel m6A reader by stabilizing c-MYC mRNA, and then promotes HGSOC progression [54].

YT521-B homology (YTH) domain-containing proteins, including YTHDF1-3, YTHDF1 and YTHDF2, have been recognized as “readers” that identify m6A modifications [55]. YTHDF1 is highly expressed in HGSOC and is related to an adverse prognosis in patients. It can act as a m6A reader by specifically binding to the m6A modification of the translation initiation factor EIF3C, augmenting EIF3C translation, and promoting OC progression [56]. Tripartite motif protein 29 (TRIM29) is aberrantly expressed in some cancers and can act as an oncogene or tumour suppressor [57]. Our research group also revealed that F-box and WD repeat domain-containing 7 (FBW7) expression is regulated in HGSOC, where it interacts and antagonizes YTHDF2 to alter the expression levels of m6A-modified Bcl-2-modifying factor (BMF) mRNA, thereby affecting cell proliferation [58].

ELAV-like protein 1 (HuR) is a member of the embryonic lethal abnormal visual system (ELAV) family. The transcriptional repressor ZEB2 not only activates EMT but also promotes the initiation and progression of cancers [59]. HuR regulates the stability and translation of ZEB2 to affect proliferation [60]. Translocase of inner mitochondrial membrane 44 (TIMM44), a peripheral membrane protein, is associated with the transport of proteins from the mitochondrial inner membrane to the mitochondrial matrix [61]. HuR modulates the expression of TIMM44 by stabilizing its mRNA levels, and its expression is correlated with OC cell progression [62]. E2F2 is a transcription activator that plays an important role in cancer progression [63]. CircE2F2 can bind to HuR to stabilize E2F2 mRNA and consequently promote OC cell proliferation, glucose metabolism and metastasis [64].

La-related protein 1 (LARP1) is a highly evolutionarily conserved RBP of the LARP family. It can regulate the stability and translation of mRNAs associated with ribosome biogenesis and cell proliferation [65]. LARP1 interacts with the 3’ UTRs of BIK (encodes a pro-apoptotic protein) and BCL2 (B-cell lymphoma 2, encodes an anti-apoptotic protein), stabilizes BCL2, and destabilizes BIK with a net effect of promoting OC proliferation [66, 67].

Heterogeneous nuclear ribonucleoproteins (hnRNPs) have been considered crucial tumour oncogenes related to proliferation and apoptosis. hnRNPA2B1 has two isoforms, hnRNPA2 and hnRNPB1, that regulate different gene expression patterns and phenotypes in various cancers [68]. HnRNPA2B1 binds to and stabilizes the transcript level of Lin28B, thereby promoting serous ovarian cancer (SOC) cells progression [69]. In addition, IGF2BP3 targets downstream human copper transporter 1 (hCTR1), induces chemoresistance and is correlated with a poor prognosis in SOC patients [70].

ESRP1, an epithelial cell RBP, participates in the EMT process by regulating alternative splicing and is associated with a poor prognosis [71]. Circ-NOLC1 promotes EOC progression by binding to ESRP1 and modulates cell-dependent kinase 1 (CDK1) and Ras homologous family member A (RhoA) expression [72].

MicroRNAs are small endogenous noncoding RNAs that regulate gene expression by promoting the degradation and/or repressing the translation of their target mRNAs. The first step in miRNA maturation is executed by the Drosha microprocessor, in which the RNase III enzyme Drosha (Drosha) and DiGeorge syndrome critical region gene 8 (DGCR8) are the core components [73, 74]. DGCR8 can recognize primary miRNAs and promote their methylation with the help of the methylase METTL3 [75]. Dorsha can also be recruited to double-stranded RNA and thereby produce precursor miRNAs [76]. The RBP DDX1 belongs to the DEAD-box helicase family, which can promote pri-miRNA maturation and repress OC progression [75].

Expression of RNA-binding motif protein 3 (RBM3), a member of the glycine-rich RNA binding protein (GRP) family, was highly upregulated in OC tissues and A2780 OC cell lines. RBM3 positively regulates the DNA damage response in OC cells and can act as a novel oncogenic prognostic biomarker for OC patients [77].

Poly C-binding protein 1 (PCBP1) can bind to the 3’ UTR of the cell cycle inhibitor p27 via its KH1 domain, thereby stabilizing p27 and inhibiting OC progression [78].

Coiled-Coil Domain Containing protein-124 (Ccdc124) is an mRNA-binding factor related to cell division and ribosome biology. Upregulated Ccdc124 expression was associated with a prolonged prognosis in OC patients [79].

The RBP of DEAD-Box Helicase 3X-Linked (DDX3X) can be stabilized by RNA Component of Mitochondrial RNA Processing Endoribonuclease (lncRNA RMRP) and promotes Phosphoglycerate dehydrogenase (PHGDH) mRNA translation, thus conferring resistance of OC cells and inhibiting OC progression [80]. Overexpression of DDX3X can reverse miR-196a-mediated OC progression by activating PTEN expression and suppressing AKT activity [81].

The plasminogen activator (PA) system plays an important role in the invasion and metastasis of OC, and PA inhibitor type 1 (PAI-1) is the main subtype [82]. PAI-RBP1 (PAI-1 mRNA-Binding Protein 1) contributes to OC progression by binding and stabilizing PAI-1 mRNA [83].

### Apoptosis

Lin28 has two paralogues, Lin28A and Lin28B. Lin28A positively binds to and upregulates the mRNA encoding Rho-associated coiled-coil containing protein kinase family 2 (ROCK2) and therefore promotes cell proliferation, invasion, and metastasis and inhibits apoptosis in OC cells [84]. LIN28B is highly expressed in HGSOC and can inhibit OC cell apoptosis by binding to AKT2 mRNA, which is associated with the DNA damage pathway, and promote its expression, regulate FOXO3A protein phosphorylation and decrease the antiapoptotic activity of BIM [85]. NEAT1 is an abundant lncRNA that has been demonstrated to be an oncogene in multiple cancers. Lin28B enhances the stability of NEAT1, whose expression is upregulated in OC cells and correlated with poor prognosis [86].

PTB, a member of the heterogeneous nuclear family (hnRNPI), regulates RNA processing and internal ribosome entry (IRES)-mediated translation [87]. PTB knockdown in OC cells inhibits cell apoptosis by alternative splicing [88].

### Invasion and epithelial-mesenchymal transcription (EMT)

Studies have revealed that IGF2BP1 can promote SRF expression in an M6A-dependent manner by impairing miRNA-directed decay [89]. These phenomena promote tumour cell growth and invasion in an SRF-dependent manner. Decreased IGF2BP2 levels significantly inhibit the proliferation of OC cells [90].

LncRNA HOX Transcript Antisense RNA (HOTAIR) is overexpressed in OC, leading to disease progression. HuR binds to the lncRNA HOTAIR, which positively regulates microRNA-373 expression and derepresses the expression of the Ras oncogene family member Rab22a, leading to the promotion of proliferation, migration and invasion [91]. It can induce paclitaxel resistance by binding to and stabilizing the mRNA encoding TUBB3 (class III b β-tubulin) in competition with miR-200c [92]. In OVCAR3 OC cells, HuR is highly overexpressed, and it binds to and stabilizes lncRNA nuclear enriched abundant transcript 1 (NEAT1), thereby positively promoting proliferation and invasion [46]. In addition, HuR contributes to tumour proliferation, migration and tumorigenicity by binding to the lncRNA metastasis-associated lung adenocarcinoma transcript 1 (MALAT1), which acts as a sponge for miR-506 and depresses the apoptosis inhibitor iASPP [93].

Gastric cancer-associated lncRNA1 (GClnc1) is a long noncoding RNA that plays an indispensable role in metastasis [94]. Forkhead box protein C2 (FOXC2) activates transcription of NOTCH1 by binding to GClnc1, thereby enhancing proliferation and EMT in OC cells [95].

Metastasis-associated lung adenocarcinoma transcript 1 (MALAT1) is a lncRNA whose expression is upregulated in OC and correlated with metastasis. Knockdown of MALAT1 expression can downregulate the splicing factor RBP Fox-1 Homologue 2 (RBFOX2) and subsequently regulate the transcription of tumour suppressor Kinesin family member 1B (KIF1B) [96].

Sorbin and SH3 domain-containing 2 (SORBS2), which is localized to the 4q35 region of the human genome, participates in signal transduction and cytoskeleton establishment [97]. Suppression of SORBS2 promotes cell migration by contributing to pseudopodia elongation and detachment of actin from focal adhesion areas [98]. SORBS2 can stabilize the tumour-suppressive immunomodulatory transcripts WAP four-disulfide core domain 1 (WFDC1) and interleukin-17D (IL-17D), thereby suppressing metastatic colonization of OC cells [99].

Our recent study indicates that CELF2 expression is positively correlated with the OS and PFS of OC patients. CELF2 increases the stability of its downstream target FAM198B by binding to AU/U-rich elements (AREs) in the 3’ UTR, modulating the proliferation, migration and invasion of OC cells in vivo and in vitro. In addition, we also demonstrate that CELF2/FAM198B can repress the progression of OC via its effects on mitogen-activated protein kinase/extracellular-regulated protein kinase (MAPK/ERK) signalling [10].

Quaking isoforms 5 (QKI5) had been implicated in the processing of microRNA (miRNA) and pre-mRNA and was downregulated in several cancers, including SOC. Studies have also demonstrated that QKI5 could inhibit metastasis by targeting transcriptional coactivator PDZ (TAZ). Mechanistically, QKI5 bound to TAZ mRNA and recruited EDC4, thus decreasing the stability of TAZ mRNA [100].

### Chemotherapy resistance

IGF2BP1 regulates chemoresistance in OC by stabilizing c-MYC and adenosine triphosphate (ATP)-dependent efflux pump MDR1 (multi-drug-resistance factor 1) [101].

Lin28B induces chemoresistance, and is correlated with a poor prognosis in HGSOC patients, by targeting downstream human copper transporter 1 (hCTR1) [70].

SFPQ (PSF, polypyrimidine tract binding protein-associated splicing factor) is a splicing factor that participates in the processes of RNA transport and apoptosis [102]. SFPQ knockdown increases platinum plus taxane-based chemotherapy (PT)-induced apoptosis by regulating alterative splicing of caspase-9 mRNA in EOC cells [103].

Cytoplasmic polyadenylation element binding protein 4 (CPEB4) has already been proven to be an RBP that contributes to transcript-level polyadenylation and translation. It binds to Taxol (paclitaxel)-resistance-associated gene-3 (TRAG-3/CSAG2), contributes to its translation, and promotes paclitaxel resistance in OC patients [104]. CSAG2 was demonstrated necessary for proliferation and tumorigenesis in vivo and CSAG2-stimulated SIRT1 activity to enhance p53 deacetylation was shown to inhibit p53 transcriptional activity, leading to improved cell survival under genotoxic stress [105]. Thus, interfering CPEB4/CSAG2 axis might be of benefit to overcome paclitaxel-resistant OC.

Y-box binding protein 1 (YBX1/YB1), a member of the cold-shock domain-containing protein family, positively regulates resistance to cisplatin and correlates with the prognosis of EOC patients [106].

RNA-binding motif protein 3 (RBM3) is associated with a favourable prognosis of OC patients and has been verified to be a positive predictor of overall survival (OS) and relapse-free survival (RFS) [107]. In addition, increased RBM3 expression promotes platinum sensitivity by regulating the apoptosis-related mediators BCL-2, BAX and DNA integrity, which are involved in DNA damage, and repairs adverse cytotoxic effects after chemotherapy [3].

### Others

Liang et al. have identified 3 significantly increased key RBPs (MRPL14, PARP4 [108] and STRAP [109]) and 3 markedly decreased RBPs (MRPL46 [110], LUC7L2 [111] and PAPOLA) related to OC by bioinformatics analysis, and subsequently verified in OC tissues by immunohistochemistry and RNA-seq data [90]. Fortunately, it is not clear how these RBPs are related to the mechanism of OC formation.

## The role of RBPs as potential diagnostic, prognostic and therapeutic biomarkers in OC

Most OC patients present with advanced abdominal pelvic metastasis and surgically unresectable disease due to a lack of reliable and valid diagnostic markers. Therefore, the early diagnosis of OC is important and is associated with a significant improvement in prognosis [64, 112].

To date, several studies have confirmed the diagnostic and prognostic roles of RBPs in different cancers, including OC [113–116]. For example, IGF2BP1 expression is mostly upregulated and correlated with poor prognosis in OC [52]. Consistently, another study using gene set enrichment analysis (GSEA) revealed that IGF2BP1 is associated with pathways and WNT signalling pathways [117]. In addition, the expression of another family member, IGF2BP3, is associated with more invasive phenotypes and poor survival rates [70]. More relevant studies are needed in the future to confirm its role as a potential diagnostic and prognostic biomarker in OC.

Next, we will review many potential therapeutic biomarkers, including antisense oligonucleotides (ASOs), small peptides, and small molecule inhibitors. In OC, 4EBP-based peptides can prevent cap-dependent translation by binding to the eIF4E factor, ultimately repressing tumour progression. They can bind to an analogue of gonadotropin-releasing hormone (GnRH), which is expressed in the majority of OC patients and has anticancer effects [118]. The synthetic peptide can prevent tumour progression without any cytotoxicity in a xenograft model of OC. In addition, fusion peptides can inhibit tumour growth by disrupting the RBM38-eIF4E interaction and thus upregulating the P53 levels with the goal of inhibiting tumour progression in vivo and in vitro [119]. Collectively, these findings support that the fusion peptide complex may be a potential therapeutic agent. In addition, microsatellite instability (MSI) is highly expressed in OC, and this high expression is associated with poor prognosis of OC patients. MSI siRNAs may be a new therapeutic strategy for reversing resistance and repressing OC tumours [120]. More relevant clinical studies are needed in the future to confirm its role as a therapeutic biomarker in OC.

## Influences of RBPs on regulating OC cellular signalling pathways

Although an increasing number of RBPs have been identified and verified, their posttranscriptional gene regulation (PTGR) signalling pathways have not been fully elucidated. It is now understood that RBPs involved in PTGR can cooperate with ncRNAs to bind to mRNAs and modify the amounts of proteins generated [5]. RBM11 can positively regulate the Akt/mTOR signalling pathway in OC cells [121]. Our previous studies have demonstrated that CELF2/FAM198B can repress the progression of OC via MAPK/ERK signalling and further be used to target the ERK pathway in the future [10]. Overexpression of LIN28B inhibits OC in vitro through regulation of the AKT2/FOXO3A/BIM axis [85]. MSI-1 effectively protects OC cells against paclitaxel treatment by ERK signalling pathway activation [120]. IGF2BP2 enhances circ0000745 and promotes aggressiveness and stemness in OC via miR-3187-3p/ERBB4/PI3K/AKT axis [122]. By TCGA analysis, down-regulation of RAD51AP1 (RAD51‐dependent homologous recombination) supressed proliferation, migration and invasion of OC cells and correlated with TGF-β/Smad pathway [123]. Under platinum and paclitaxel (PT) treatment, SFPQ/p54^nrb^/SRSF2 pathway plays a crucial role in OC resistance [103].

## Future perspectives and conclusions

Due to the late diagnosis and high rate of relapse, OC patients have poor prognosis, and its specific pathogenesis of OC is still unclear. Therefore, it is of outer importance to identify earlier diagnostic and more efficient therapeutic approaches for clinical application of OC. Currently, increasing evidence reveals that changes in RBP expression affect multiple steps of OC progression. In this review, RBPs were revealed to participate in multiply biological processes of OC and can serve as promising biomarkers for the diagnosis, prognosis, and therapy of OC. At present, a few studies have already provided proof-of-concept evidence regarding the in vitro use of small-molecule inhibitors, therapeutic peptides or ASOs to selectively antagonize RBPs or RBP-RNA interactions, as verified by experiments using eIF4E, MSI, and LIN28, which have shown favourable functional outcomes [118–120, 124]. Nevertheless, whether RBPs can successfully act as effective biomarkers for the diagnosis, prognosis and therapy of OC is still far from clinical application. The application of specific RBPs related to human disease in the treatment of tumours is the ultimate goal of RBP-related research.

Therefore, some suggestions are put forward for future research on RBPs in OC. Firstly, although many researchers have initially demonstrated some mechanisms of RBPs’ dysregulated expression. More scientific researches and explorations are indispensable needed to fully clarify the mechanism of biogenesis of RBPs. A complete annotation of RBP dysregulated expression will undoubtedly enhance our understanding of its function. Secondly, RBPs exert their functions by binding to downstream RNA, forming RNP complexes and subsequently regulate the functions of RNA transcripts via multiple posttranscriptional mechanisms. Even small alterations in the expression can cause significant changes in regulatory networks. Therefore, it is imperative to elucidate other further mechanisms that we do not know. Thirdly, the detection of RBPs is currently mainly applied in tumor tissues. Thus, the expression of RBPs should be detected in more clinical samples, such as blood, urine. Combined detection methods should be exploited to obtain more diagnostic values. Fox example, special RBPs could be combined with traditional detection markers to improve the sensitivity and specificity of disease diagnosis. Fourthly, how to deliver RBPs to the tumour sites of the body, how to avoid the immune responses, how to ensure stable and effective function are difficult problems that need be addressed urgently.

In summary, our current understanding of RBP functions in OC is still very limited. Fortunately, with the accelerated development of biotechnology and bioinformatics analyses, more RBPs will be discovered and validated. In the near future, we believe that in-depth knowledge of RBPs and effective application of RBPs in clinical practice will represent a giant breakthrough in the treatment of OC.

## Data Availability

Not applicable.
